# Femtosecond Raman-induced Kerr effect spectroscopic study of the intermolecular dynamics in aqueous solutions of imidazolium hydrochloride, imidazole, sodium triazolide, and triazole: concentration dependence

**DOI:** 10.1007/s44211-024-00692-7

**Published:** 2024-11-20

**Authors:** Masako Shimizu, Hideaki Shirota

**Affiliations:** Department of Chemistry, Chiba University, 1-33 Yayoi, Inage-Ku, Chiba, 263-8522 Japan

**Keywords:** Raman-induced Kerr effect spectroscopy, Low-frequency spectrum, Intermolecular vibration, Orientational relaxation, Aromatics, Aqueous solution

## Abstract

In this study, we employed femtosecond Raman-induced Kerr effect spectroscopy to analyze the concentration-dependent intermolecular dynamics in positively or negatively charged aromatics and their neutral analogous aromatics (imidazolium hydrochloride (ImHCl), imidazole (Im), sodium triazolide (NaTr), and triazole (Tr)) in aqueous solutions at 293 K. We also measured their liquid properties, such as density, viscosity, and surface tension, at 293 K, and compared them with their dynamic properties. Furthermore, we performed the quantum chemistry calculations of the target aromatics and some clusters to elucidate their optimized structures, interaction energies, charge populations, and Raman-active normal modes. We characterized the Kerr transients over 2 ps using a triexponential function. The results revealed that the aqueous solutions’ intermediate and slow relaxation time constants were linearly proportional to the viscosities. The slopes of the time constants to the viscosity of the aqueous ImHCl solutions were steeper than those of the aqueous Im solutions, whereas the slopes of the aqueous NaTr solutions were milder than those of the aqueous Tr solutions. These findings indicated that the charge of the aromatics in the aqueous solutions affected the coupling parameter between the solute and solvent in the orientational dynamics with different ways. The first moment (*M*_1_) of the low-frequency band (< 200 cm^−1^), coming from the intermolecular vibrations, in the difference spectra between the aqueous aromatic solutions and neat water shifted to the high-frequency region as the concentration increased. The *M*_1_ slope to the concentration for the aqueous ImHCl solutions was steeper than that for the aqueous Im solutions. Conversely, the concentration dependence of *M*_1_ for the aqueous NaTr solutions was similar to that for the aqueous Tr solutions. We used the local structures of the target aromatics based on the quantum chemistry calculations to rationally clarify their concentration-dependent intermolecular dynamics in the aqueous solutions.

**Graphical abstract:**

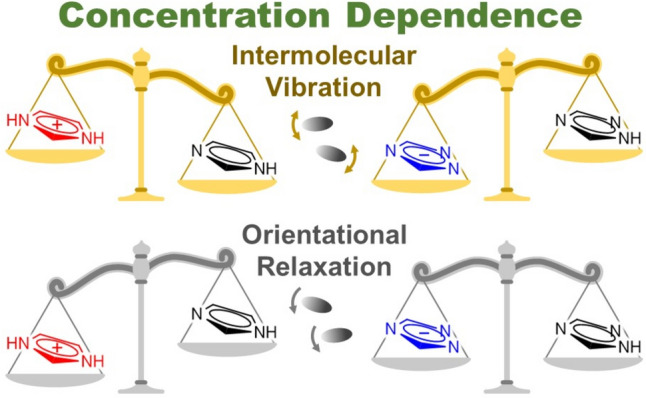

**Supplementary Information:**

The online version supplementary material available at 10.1007/s44211-024-00692-7.

## Introduction

The intermolecular dynamics of solutes in solutions are crucial to understanding the chemical reactions and transportation processes in solutions, as solute dynamics is coupled with the solvent motions [1–7]. The intermolecular dynamics in liquids and solutions comprise reorientation, interaction-induced motion, and libration. Theses molecular motions’ time scales (or frequency regions) are typically between 100 fs and 100 ps (300–0.3 cm^−1^), depending on the liquid/solution. Femtosecond Raman-induced Kerr effect spectroscopy, fs-RIKES, is an effective technique for observing the low-frequency spectra of liquids and solutions [8–12]. This technique is also referred to as the femtosecond optical Kerr effect spectroscopy. Notably, fs-RIKES is a third-order nonlinear spectroscopic technique using a femtosecond pulse laser. Its primary advantage lies in its low-frequency-spectrum-obtaining capability without the contribution of the Rayleigh scattering. Typical fs-RIKES under a depolarized-signal polarization condition provides low-frequency spectra with frequency ranges of 0.5–500 cm^−1^, which completely covers the intermolecular vibrational motions as well as collective orientational dynamics of conventional aprotic molecular liquids and solutions having relatively less viscosity (*η*). Thus far, fs-RIKES has been applied to the elucidation of the intermolecular dynamics of simple molecular liquids and solutions [13–17], as well as various complex systems [18], including biomolecules in solutions [19–23] and films [24], biopolymers in solutions [25], bioanalogous systems [26–28], aqueous drug solutions [29], microemulsions [30, 31], polymer liquids [32] and solutions [33–38], solvents in nanoconfined glasses [39, 40], aqueous and nonaqueous salt solutions [41–44], ionic liquids (ILs) [45–51], hydrated ILs [52], and deep eutectic solvents [53–55].

Generally, the aromatic ring libration significantly contributes to the low-frequency region (< 150 cm^−1^) depolarized Raman-spectral density of aromatic liquids, including neutral molecular liquids [15, 17, 56], as well as ILs [46, 49, 57, 58]. A typical example of such liquids is benzene [59–70]. The line shape of the low-frequency spectrum due to the intermolecular vibration of liquid benzene is trapezoidal, having a gentle peak at approximately 50 cm^−1^, with a spectral width of ~ 100 cm^−1^. Stratt and coworkers’ detailed molecular dynamics (MD) simulation study of liquid benzene revealed the dominant contribution of aromatic ring libration to the low-frequency-depolarized Raman spectrum (*I*_DRS_(*ω*)) [71, 72]. Further, Ratajska-Gadomska used theoretical calculations to demonstrate the impact of a momentary quasicrystalline order of benzenes on the low-frequency spectrum [73].

An example of the fascinating behaviors of the low-frequency spectrum of aromatic liquids and solutions might be determined by comparing the low-frequency *I*_DRS_(*ω*) of ionic (or charged) and neutral aromatic molecular systems. For example, a fs-RIKES-based comparative study of the low-frequency *I*_DRS_(*ω*) of 1-methoxyethylpyridinium dicyanamide (an IL) and an equimolar mixture of 1-methoxyethylebenzene and dicyanmethane (neutral analogues with isoelectronic structures) revealed that the low-frequency spectrum of 1-methoxyethylpyridinium dicyanamide appeared at a higher-frequency region (magnitude: 13 cm^−1^) than that of its analogous neutral binary mixture [74]. Further, the comparisons of the low-frequency spectra of ILs bearing neutral- or charged-aromatic ring cations (e.g., 1-benzyl-1-methylpyrrolidinium and 1-cyclohexylmethyl-3-methylimidazolium cations) elucidated that the aromatic ring libration of the positively charged IL-contained aromatic ring proceeded faster than that of its similar neutral IL-contained aromatic ring [75, 76].

**Fig. 1:**
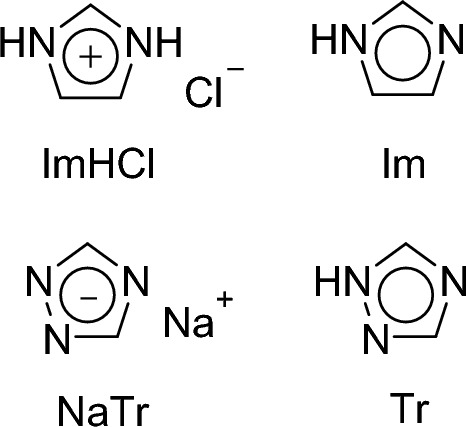
Chemical formulas of ImHCl, Im, NaTr, and Tr

We recently compared the low-frequency spectra of positively and negatively charged aromatics (imidazolium hydrochloride (ImHCl) and sodium 1,2,4-triazolide (NaTr) salts, respectively, Fig. 1) with those of their isoelectronic-structured neutral aromatics (imidazole (Im) and 1,2,4-triazole (Tr), Fig. 1) in aqueous solutions via fs-RIKES [77]. We observed that the peak or shoulder in the high-frequency region (> 60 cm^−1^) of the low-frequency broadened band of the aqueous solution of ImHCl exhibited a higher frequency than that of Im, whereas that of NaTr exhibited a lower frequency than that of Tr. The results obtained from that study further clarified that the high-frequency region of the low-frequency broadened band of the aqueous solutions of ImHCl and Im with a concentration of 5.0 mol dm^−3^ exhibited a redshift with the increasing temperature, whereas that of the same solutions with a concentration 1.0 mol dm^−3^ did not exhibit any temperature-dependent change [77].

In this study, we further explored the detailed concentration dependence of the intermolecular dynamics in aqueous solutions of ImHCl, Im, NaTr, and Tr using fs-RIKES. This study was primarily conducted to elucidate the effects of the charges on aromatics in aqueous solutions on their concentration-dependent low-frequency dynamics, including their intermolecular vibrations and orientational relaxations. After that, we discussed the results of the spectroscopic study based on the physical properties, such as the *η*, density (*ρ*), and surface tension (*γ*) of the explored solutions. Furthermore, we clarified the concentration dependence of the intermolecular dynamics in the aqueous aromatic solutions via the quantum chemistry calculations of the optimized structures, charge populations, and Raman-active normal modes of the aromatics and some clusters.

## Experimental methods

ImHCl (Aldrich, > 98%), Im (Kanto, > 98%), NaTr (Combi-Blocks, > 97%), Tr (TCI, > 99%), and water (Wako, ultrapure, liquid chromatography/mass spectrometry grade) were used as received. The aqueous solution samples were prepared at various concentrations (*c*) from 0 mol dm^−3^ (neat water) to the near-saturated concentration of each sample (5.0, 10.0, 2.5, and 5.0 mol dm^–3^ for ImHCl, Im, NaTr, and Tr, respectively).

Regarding the liquid properties measurements, the *ρ* values of the sample solutions were obtained using a temperature-controlled densitometer (Anton Paar, DMA 4100 M) at 293.0 ± 0.1 K; the *η* values were determined using a reciprocating electromagnetic piston viscometer (Cambridge Viscosity, ViscoLab 4100) with a constant temperature of 293.0 ± 0.2 K maintained by a circulating water bath (Yamato, BB300). Further, the *γ* values of the sample solutions were measured at 293.0 ± 0.2 K using a contact-angle meter (Kyowa Interface Science, DMs-401) equipped with a circulating water bath (EYELA, NCB-1210).

A laboratory-built fs-RIKES apparatus was used to obtain the low-frequency spectra in the 0.3–700 cm^–1^ frequency range, and the details of the fs-RIKES have been reported elsewhere [17, 63]. Briefly, a titanium sapphire laser (KMLabs Inc., Griffin) was used as the light source for the most current fs-RIKES setup [78]. This laser was pumped by the second-harmonic light of an Nd:YVO_4_ diode laser (Spectra Physics, Millennia Pro 5sJ), and the output power was ca. 420 mW. The typical temporal response of the fs-RIKES setup was ca. 37 ± 3 fs (full-width at half-maximum); it was estimated from the cross-correlation between the pump and probe pulses using a potassium dihydrogen phosphate crystal (type I, thickness: 200 µm). To obtain the depolarized Raman signal, the pump and probe light polarizations were set at 90° and + 45°, and the analyzer was set at − 45°. For each aqueous solution, a high time resolution of 3.335 fs/step with 2048 points scans was obtained from − 1.0 to 5.8 ps. For neat water, scans were obtained from − 1.0 to 10.0 ps at 3.335 fs/step. Further, long-time-window data were obtained at 33.35 or 66.71 fs/step, and the total points depended on the sample solution and concentration. To obtain the averaged data, three- and five-time scans were performed for the high-time-resolution (short-time-window) and long-time-window (low-time-resolution) measurements, respectively. Pure heterodyne signals were obtained and used to eliminate residual homodyne contributions by combining the Kerr transients measured at + 1.5° and − 1.5° rotations of the input polarizer. A Peltier temperature controller (Quantum Northwest, Luma 40) was used to maintain the sample temperature at 293 K. Furthermore, the sample solutions were injected into a 3 mm optical-path-length quartz cell (Tosoh Quartz) using 0.2 and 0.02 µm pore-size Anotop filters (Whatman) before the fs-RIKES measurements.

A steady-state Raman (ss-Raman) spectrometer was employed to measure the Raman spectra in the relatively high-frequency range; the details of the apparatus have been reported elsewhere [77]. Briefly, a diode laser (RGB Lasersystems, Lambda Beam 785-225WL) with an output power of 225 mW at 784.5 nm and the vertical polarization, was used as the light source. The polarizer was set at 90° to the incident light to obtain *I*_DRS_(ω). A spectrometer (Andor, SR-500i-B1-R) and a charge-coupled-device camera (Andor, DU420A-BEX2-DD) were used to obtain the Stokes Raman spectra. Neon and halogen lamps were also used to calibrate the wavelength and intensity, respectively. As aforementioned, the temperature of the sample was maintained at 293 K using a Peltier temperature controller (Quantum Northwest, Luma 40). Before performing the ss-Raman measurements, the sample solutions were injected into a 10 mm optical-path-length quartz cell (Tosoh Quartz) using 0.2 and 0.02 µm pore-size Anotop filters (Whatman).

In addition, the gas-phase optimized structures of Im^+^, Im, Tr^−^, and Tr and some of their clusters (Im^+^–Cl^−^, Tr^–^–Na^+^, and Tr–Tr) were calculated based on the MP2/aug-cc-pVTZ level of theory using the Gaussian 16W program suite [79]. The intermolecular interaction energies of the clusters were also calculated using the counterpoise method [80, 81]. The atomic charges (charge distribution) of the four aromatics (ImHCl, Im, NaTr, and Tr) were characterized based on the natural bond orbital (NBO) analysis [82–84]. Though the experimental condition in this study is the solution, the gas phase calculations are helpful to discuss a qualitative picture of the experimental results.

## Results

### Density, viscosity, and surface tension

**Fig. 2:**
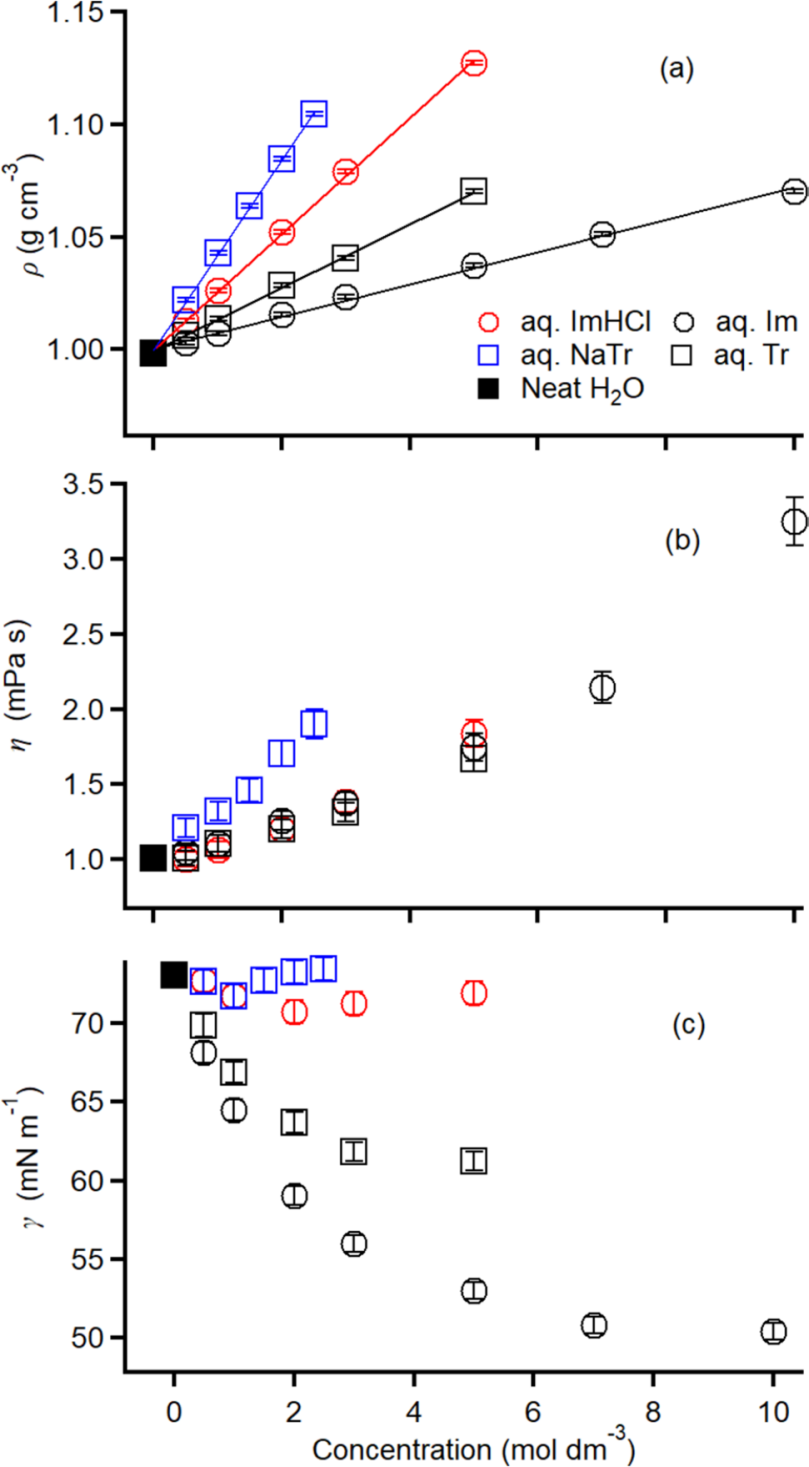
Concentration dependence of **a** *ρ*, **b** *η*, and **c** *γ* of the aqueous solutions of ImHCl (red circles), Im (black circles), NaTr (blue squares), and Tr (black squares) at 293 K. Filled black squares show the data of the utilized neat water

Figure 2a shows the *ρ* vs. *c* plots of the aqueous solutions of ImHCl, Im, NaTr, and Tr, as well as neat water. As observed, *ρ* was linearly proportional to *c* in the four solutions, although each solution series displayed a unique slope: 26.2 × 10^3^, 7.43 × 10^3^, 43.0 × 10^3^, and 14.4 × 10^3^ g mol^–1^ in the aqueous solutions of ImHCl, Im, NaTr, and Tr, respectively. Notably, the aqueous solutions of the salts exhibited larger proportional constants than those of the corresponding neutral aromatics.

Figure 2b shows the concentration dependence of *η* in the aqueous solutions of ImHCl, Im, NaTr, and Tr. The *η* values of the four aqueous solutions increased with the increasing concentration. Further, *η* of the aqueous NaTr solution exhibited the highest concentration sensitivity among the four solutions; the other three solutions displayed similar concentration dependence of *η*.

Figure 2c shows the *γ* vs. *c* plots of the aqueous solutions of ImHCl, Im, NaTr, and Tr. Notably, the *γ* values of the aqueous solutions of ImHCl and NaTr remained almost the same with varying *c* values. In contrast, those of the aqueous solutions of the neutral aromatics decreased with increasing concentration.

The data of the liquid-state properties (*ρ*, *η*, and *γ*) of the aqueous aromatic solutions at 293 K are summarized in Table S1.

### Orientational relaxation

**Fig. 3:**
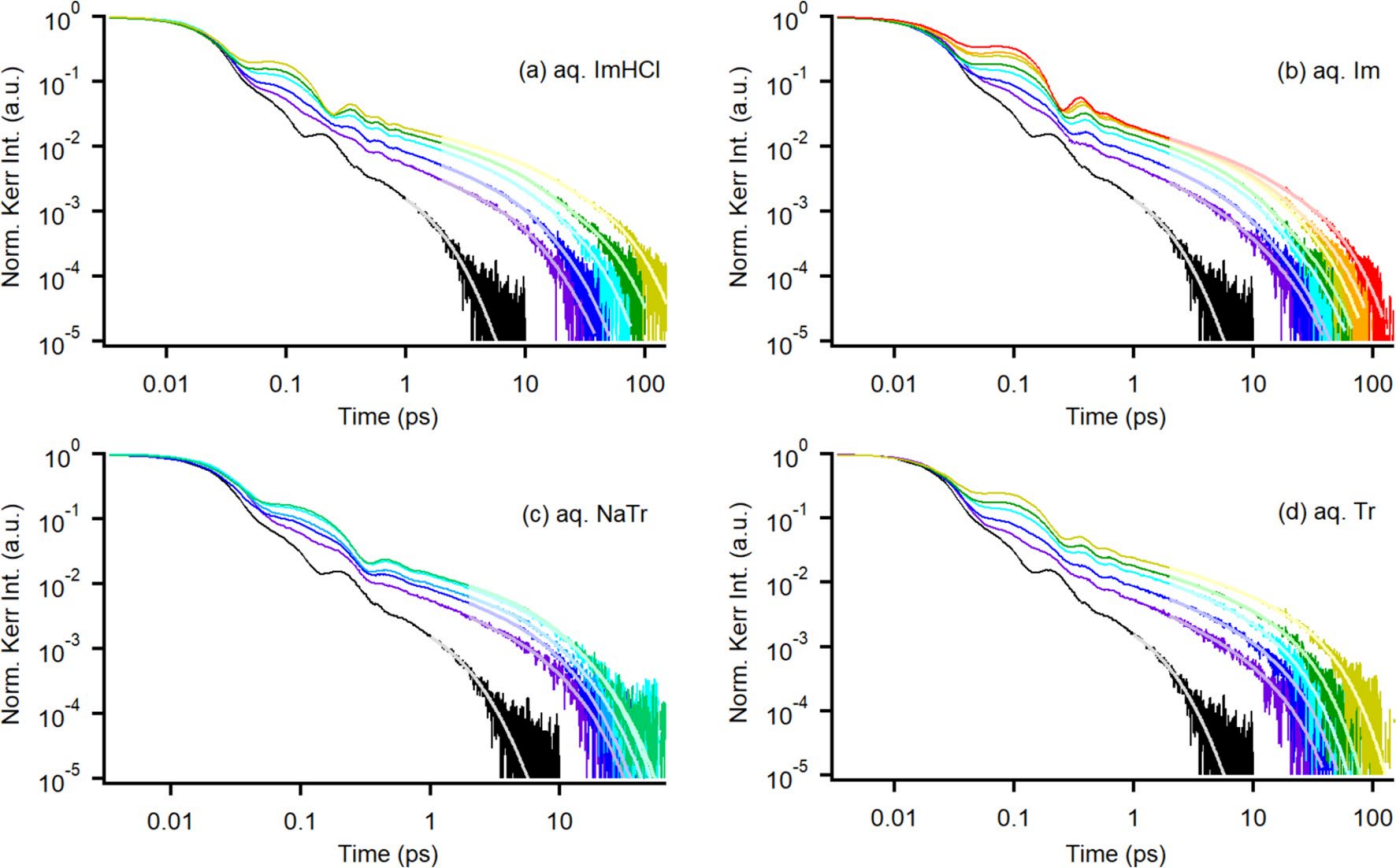
Logarithmic plots of the Kerr transients of various-concentration aqueous solutions of **a** ImHCl, **b** Im, **c** NaTr, and **d** Tr (0.5 mol dm^−3^: purple, 1.0 mol dm^−3^: blue, 1.5 mol dm^−3^: light blue, 2.0 mol dm^−3^: cyan, 2.5 mol dm^−3^: light green, 3.0 mol dm^−3^: green, 5.0 mol dm^−3^: yellow, 7.0 mol dm^−3^: orange, and 10.0 mol dm^−3^: red). A black solid line represents the Kerr transient of neat water as a reference. Multiexponential fits are also shown

**Fig. 4:**
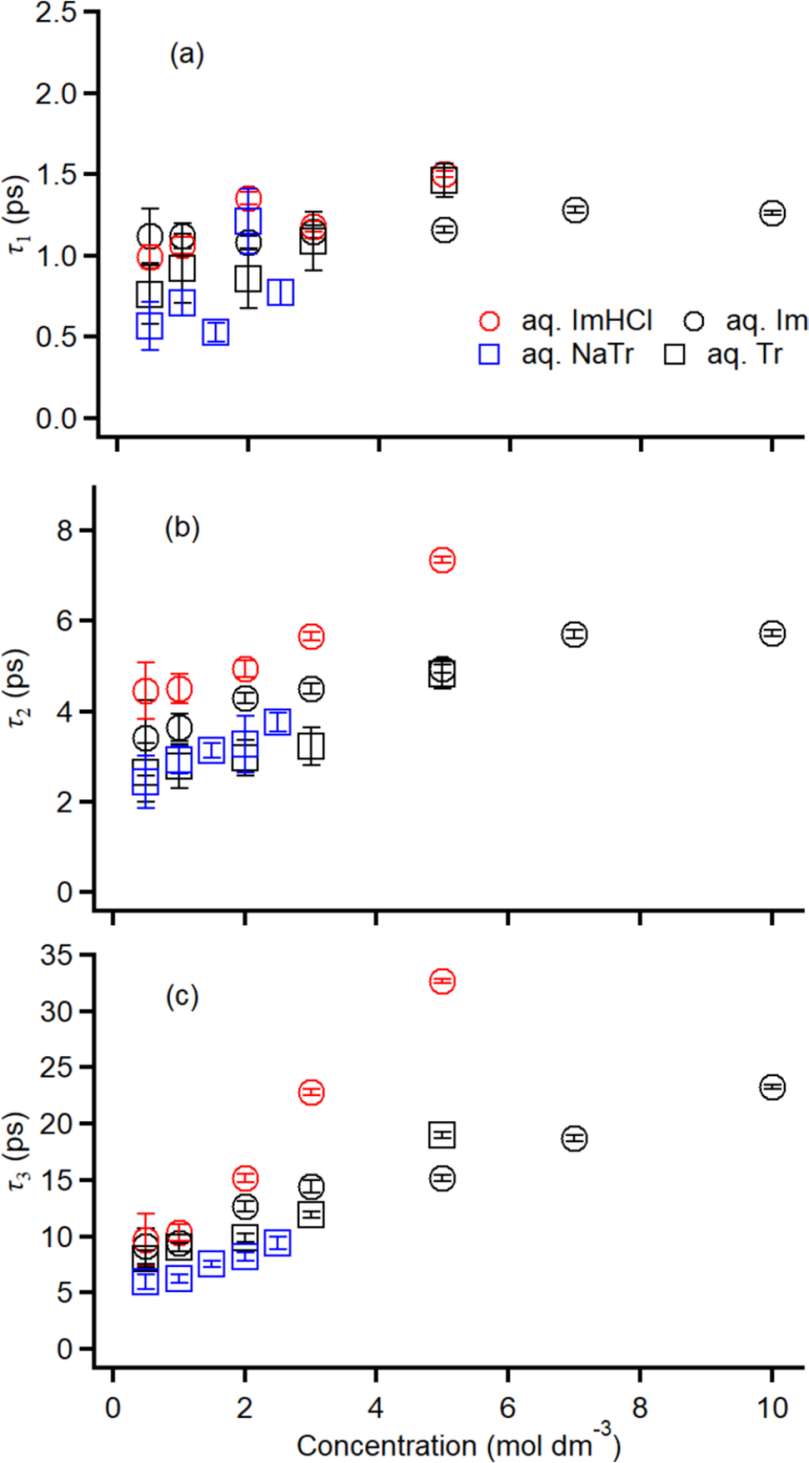
Plots of **a** fast relaxation time *τ*_1_, **b** intermediate relaxation time *τ*_2_, and (3) slow relaxation time *τ*_3_ against *c* for the aqueous solutions of ImHCl (red circles), Im (black circles), NaTr (blue squares), and Tr (black squares)

Figure 3 shows the logarithmic plots of the Kerr transients that were normalized at the *t* = 0 intensity (electronic response) for various concentrations of aqueous ImHCl, Im, NaTr, and Tr solutions; moreover, the Kerr transient of neat water is shown as a reference. The Kerr intensity proceeding from the nuclear response relative to the electronic response in the aqueous solutions of the aromatics increased with the increasing *c*. Additionally, the underdamped Kerr transient feature (observed in the time region below ~ 1 ps) became clearer as *c* increased. Furthermore, the overdamped relaxation decay became slower with the increasing *c*. These concentration-dependent Kerr transient features of the aqueous solutions have been observed in the aqueous solutions of various solutes, such as simple polymers [33–35], small molecular and ionic solutes [28, 41–43, 77], biomolecules [19–23], and drug molecule [29]. The overdamped Kerr transients of over 2 ps in the aqueous aromatic solutions were analyzed using a triexponential function, except for neat water (the fit was obtained for over 1 ps using a biexponential function). A biexponential function was used for the Kerr transients of aqueous aromatic solutions with concentrations of 1.0 mol dm^−3^ in our previous study [77]; however, we confirmed that a triexponential function ensured a better quality fit for the aqueous aromatic solutions with various concentrations, especially for the high concentration samples. Table S2 summarizes the fit parameters deployed in this study. As shown in Fig. 4, the relaxation times were plotted against *c*. As the time required by the fast relaxation component (*τ*_1_) was similar to the relaxation time of neat water and was less dependent on the concentration compared with the times required by the intermediate and slow relaxation components, we consider the intermediate and slow relaxation components are coming from the orientational dynamics of the aromatics, not bulk-like water, in this study.

### Broadband low-frequency Raman spectrum

**Fig. 5:**
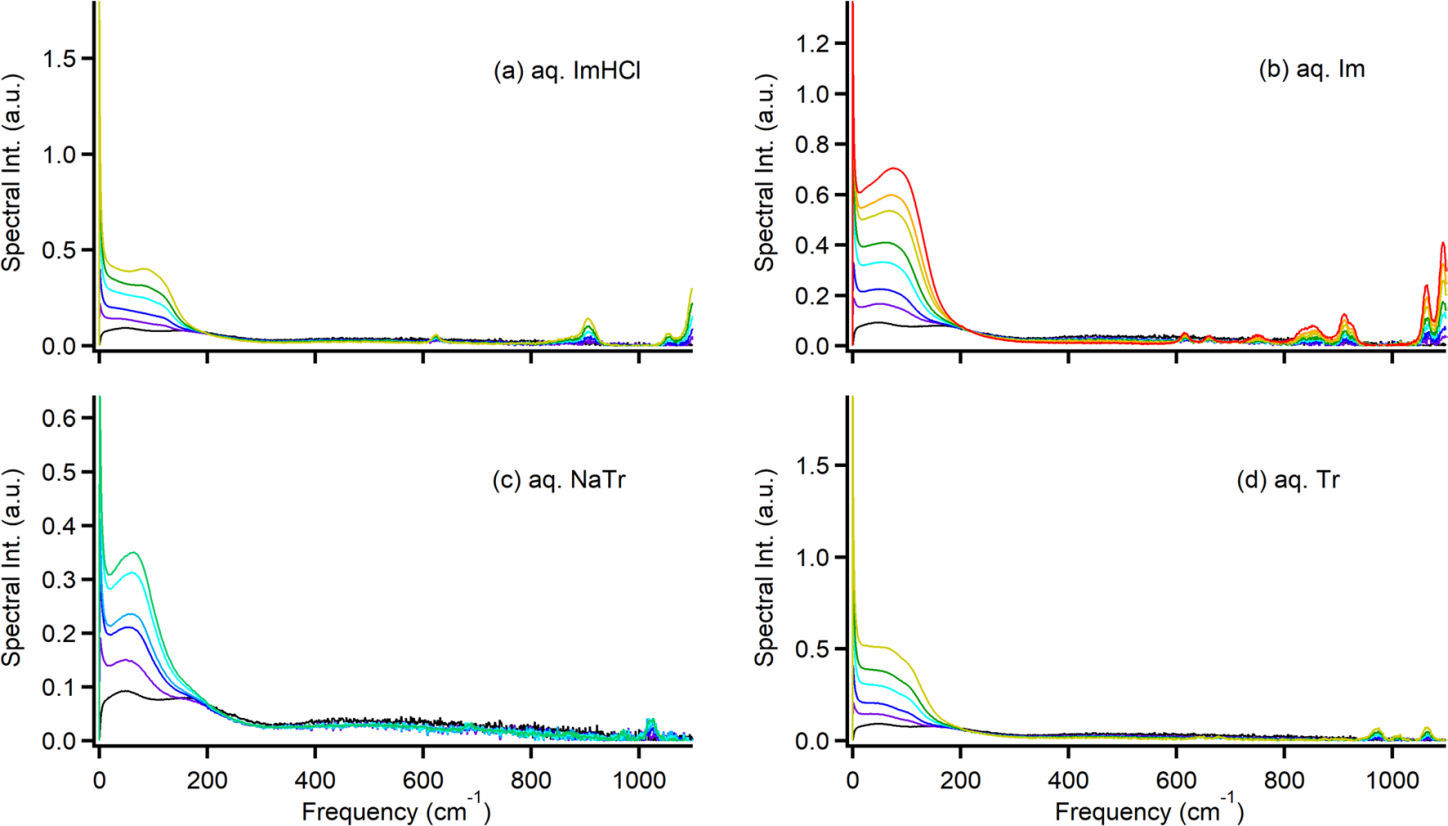
Broadband *I*_Kerr_(*ω*) of various-concentration aqueous solutions of **a** ImHCl, **b** Im, **c** NaTr, and **d** Tr (0.5 mol dm^−3^: purple, 1.0 mol dm^−3^: blue, 1.5 mol dm^−3^: light blue, 2.0 mol dm^−3^: cyan, 2.5 mol dm^−3^: light green, 3.0 mol dm^−3^: green, 5.0 mol dm^−3^: yellow, 7.0 mol dm^−3^: orange, and 10.0 mol dm^−3^: red). Raman spectrum of neat water is represented by a solid black line as a reference

The Kerr transients obtained by fs-RIKES were converted into Kerr spectra (*I*_Kerr_(*ω*, imaginary part) via Fourier transform deconvolution analysis, which was developed in detail by McMorrow and Lotshaw [85, 86]; the details of the procedure performed in this study have been reported elsewhere [17, 63]. As water exhibits a very broad intermolecular vibrational band [87–90], much broader than *I*_Kerr_(*ω*) measured in this study, we also recorded *I*_DRS_(*ω*) of the sample aqueous solutions via ss-Raman spectroscopy and connected *I*_Kerr_(*ω*) to the ss-Raman spectrum via fs-RIKES; the details of the spectrum patching procedure have been presented elsewhere [77]. As the ss-Raman spectrum in this study was detected as the signal of the Stokes scattering, and *I*_Kerr_(*ω*) via fs-RIKES includes both Stokes and anti-Stokes scatterings, *I*_DRS_(*ω*) via ss-Raman spectrometer was multiplied by the Bose–Einstein thermal occupation factor [89, 91].

1 $$I_{{{\text{Kerr}}}} \left( \omega \right) = I_{{{\text{DRS}}}} \left( \omega \right)\left[ {1 - {\text{exp}}\left( { - \hbar \omega /k_{{\text{B}}} T} \right)} \right]/\hbar^{2}$$

where $\hslash$ is the reduced Planck constant (*h*/2π) and *k*_B_ is the Boltzmann constant. The Kerr and ss-Raman spectra were optimized at the 150–250 cm^−1^ frequency range and patched at 200 cm^−1^ to obtain the broadband Bose–Einstein corrected *I*_DRS_(*ω*) (hereafter known as the broadband Raman spectrum) with a frequency range of 0.3–1100 cm^−1^. Figure 5 shows the broadband Raman spectra of the aqueous aromatic solutions with various concentrations. The spike near 0 cm^−1^ is not due to the Rayleigh scattering; it stemmed from the orientational dynamics. Figure S1 shows the semilogarithmic plots of the spectra to clarify the low-frequency region. The low-frequency region (< 300 cm^−1^) broadened bands, except for the spike near 0 cm^−1^, in this study were due to intermolecular vibrations, such as librations (rotational motions) and interaction-induced motions (translational motions) [71, 77]. A broad band from 300 to 1000 cm^−1^ for neat water was due to the libration [88–90, 92]. Thus, the band was observed in the aqueous aromatic solutions; its intensity decreased with the increasing *c* of the aqueous solutions.

**Fig. 6:**
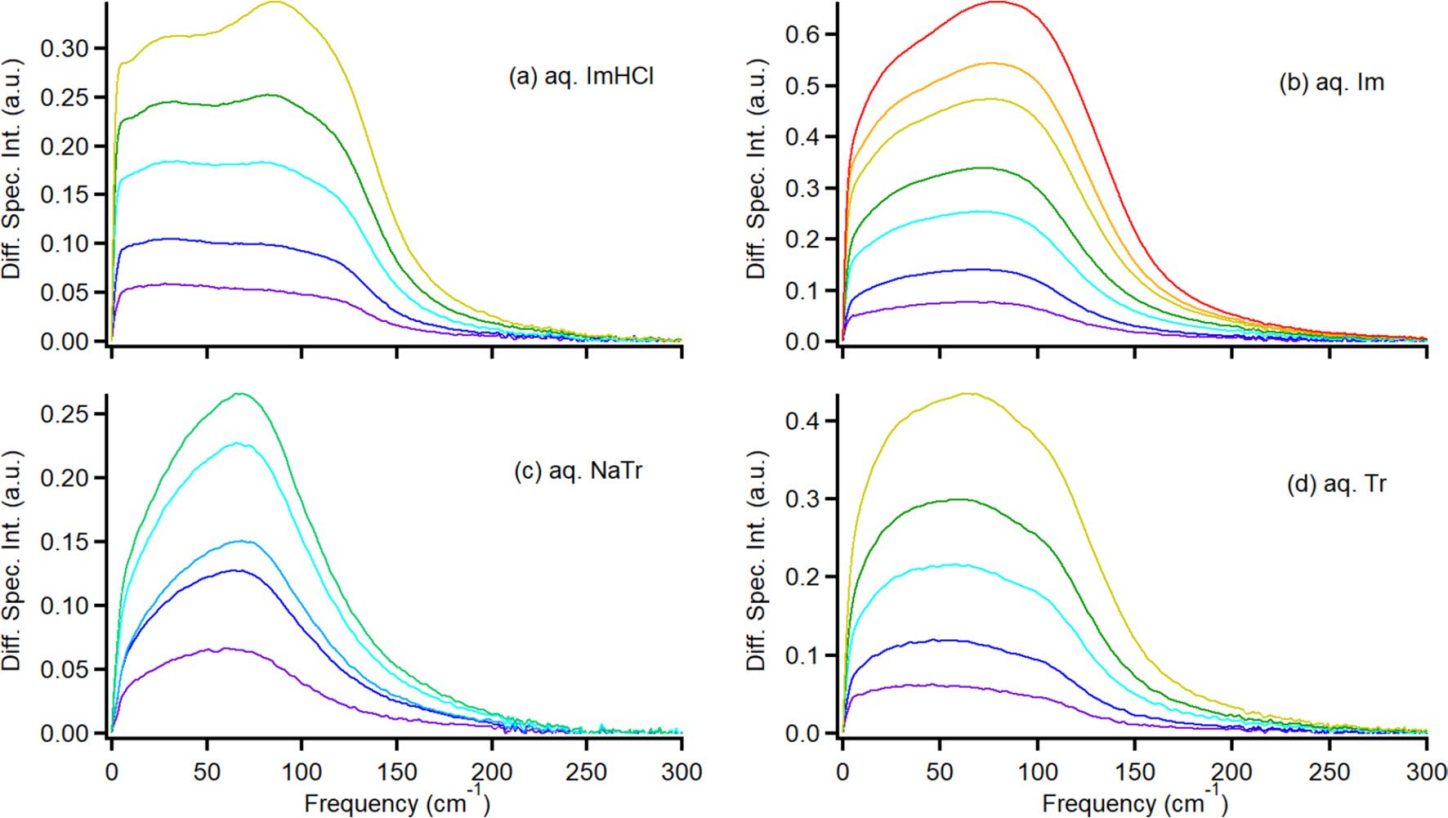
Difference low-frequency *I*_Kerr_(*ω*) of various-concentration aqueous solutions of **a** ImHCl, **b** Im, **c** NaTr, and **d** Tr aqueous solutions (0.5 mol dm^−3^: purple, 1.0 mol dm^−3^: blue, 1.5 mol dm^−3^: light blue, 2.0 mol dm^−3^: cyan, 2.5 mol dm^−3^: light green, 3.0 mol dm^−3^: green, 5.0 mol dm^−3^: yellow, 7.0 mol dm^−3^: orange, and 10.0 mol dm^−3^: red) to neat water

In this study, we subtracted the broadband *I*_Kerr_(*ω*) of neat water from that of the aqueous aromatic solutions to obtain the difference Raman spectrum, which is the spectrum of the aqueous solution without the contribution of “bulk-like” water. The broadband Raman spectra of the aqueous aromatic solutions and neat water were optimized at the 300–800 cm^−1^ frequency range (librational band), after which the difference was determined. Figure 6 shows the low-frequency difference spectra of the various concentration aqueous aromatic solutions relative to those of the neat water. Notably, the contributions of the picosecond (ps) orientational dynamics (expressed by the intermediate and slow exponential components) were also removed from the spectra to focus on the intermolecular vibrational bands of the aromatics.

### Quantum chemistry calculations

**Fig. 7:**
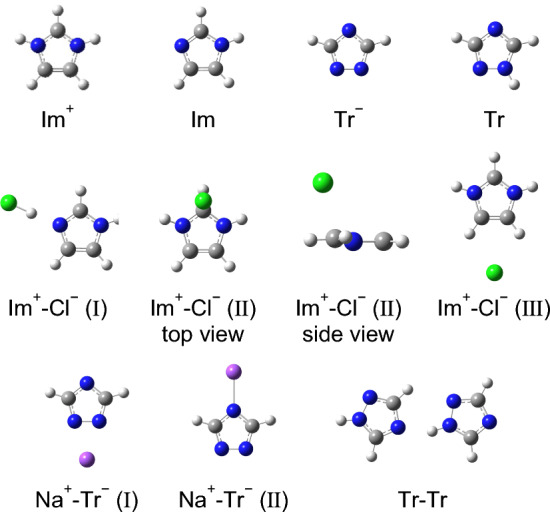
Optimized structures of the aromatics, counterions, and clusters and locally stabilized structures of the clusters at the MP2/aug-cc-pVTZ level of theory

Figure 7 shows the optimized structures of the aromatics, counterions, and clusters at the MP2/aug-cc-pVTZ level of theory. The clusters also exhibited several local minimum structures. Table S3 summarizes the atomic coordinates of the calculated aromatics and clusters. Although a wide concentration range of aqueous salt solutions includes not only free ion and contact ion pair [93], but also solvent-shared ion pair, etc., we consider the two extreme conditions, free ion or molecule and contact ion pair (and neutral dimer, Tr–Tr), in the present quantum chemistry calculations. The Na^+^–Tr^−^ cluster shows two stabilized structures (Types I and II). Both the structures are in-plane conformations. Im^+^–Cl^−^ clusters exhibited in-plane (Types I and III) and out-of-plane (Type II) stable structures. The optimized structure of the Tr dimer was made via a hydrogen bond. The energies and the interaction energies, of the aromatics, counterions, and clusters are listed in Table 1.

**Table 1:** Energies of the Aromatics, Counterions, and Clusters and the Interaction Energies of the Clusters Optimized based on the MP2/aug-cc-pVTZ Level of Theory

Aromatics/Counterions	Energy(kJ/mol)	Cluster	Energy (kJ/mol)	Interaction Energy (kJ/mol)
Im^+^	− 593,786.30	Im^+^–Cl^−^ (I)	− 1,801,415.68	− 789.69
Im	− 592,821.92	Im^+^–Cl^−^ (II)	− 1,801,340.68	− 414.93
Tr^−^	− 633,459.94	Im^+^–Cl^−^ (III)	− 1,801,297.25	− 360.54
Tr	− 634,912.84	Na^+^–Tr^−^ (I)	− 1,058,478.38	− 539.57
Na^+^	− 424,480.39	Na^+^–Tr^−^ (II)	− 1,058,408.47	− 470.24
Cl^−^	− 1,207,154.47	Tr–Tr	− 1,269,867.14	− 43.22

**Fig. 8:**
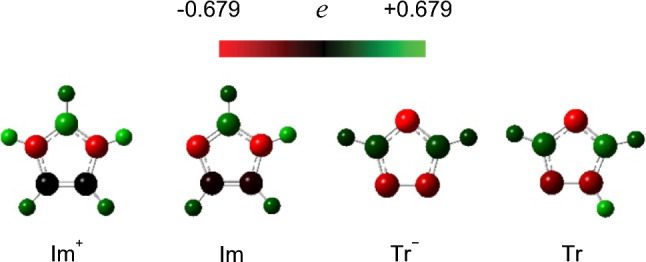
Charge populations based on the NBO analyses of the aromatics optimized at the MP2/aug-cc-pVTZ level of theory

Figure 8 shows the illustrations of the atomic charges of the four aromatics based on the NBO analyses. If we take the structure and charge population of Im^+^ into the consideration, the locally stabilized structure of Im^+^–Cl^−^ (II) (out-of-plane) seems reasonable. Additionally, the charge population of Tr indicated a possible complex formed via hydrogen bonding between NH and N(4) as an optimized structure of Tr dimer (Fig. 7).

## Discussion

### Orientational relaxation

**Fig. 9:**
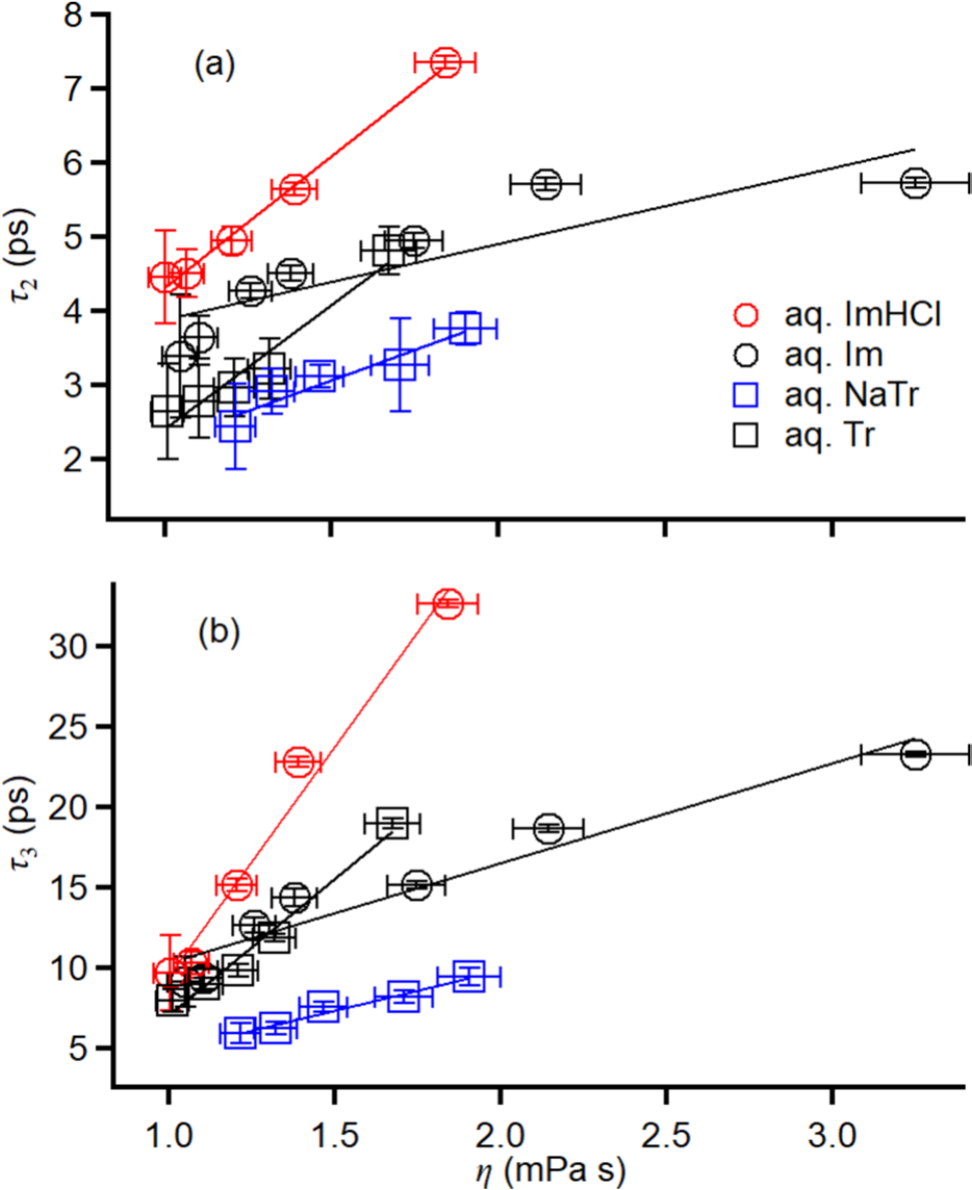
Plots of **a** *τ*_2_ and **b** *τ*_3_ vs. *η* for the aqueous solutions of ImHCl (red circles), Im (black circles), NaTr (blue squares), and Tr (black squares). Linear fits are also shown

Based on the Stokes–Einstein–Debye (SED) model, the rotational time (*τ*_s_) of a single spherical solute in a solution is expressed as follows:

2 $$\tau_{{\text{s}}} = \begin{array}{*{20}c} {\eta_{m} V} \\ {\overline{{k_{B} T}} } \\ \end{array} ,$$

where η*η*_*m*_ is the viscosity of the medium, *V* is the volume of the solute_,_ and *T* is the absolute temperature, respectively [94, 95]. Our fs-RIKES experiments observed the “collective” orientational relaxations in the liquids and solutions. The collective rotational time (*τ*_c_) is related to *τ*_s_ as follows [95, 96]:

3 $$\tau_{{\text{c}}} = \begin{array}{*{20}c} {g_{2} } \\ {\overline{{j_{2} }} } \\ \end{array} \tau_{{\text{s}}} ,$$

where *g*_2_ is the static orientation pair correlation parameter, which depends on the solution/liquid system, and *j*_2_ is the dynamic orientation pair correlation parameter, often treated as unity. Thus, the SED model promotes understanding the qualitative features of the orientational relaxation time. As the fixed temperature in this study was 293 K, *η* accounted for the dominant and essential factor that determined the orientational relaxations in the explored solutions. Figure 9 shows the plots of *τ*_2_ and *τ*_3_ vs. *η* for the aqueous aromatic solutions. Each aqueous solution system exhibited a linear relation between the relaxation time (for *τ*_2_ and *τ*_3_) and *η*, i.e., *τ*_*n*_ = *τ*_*n*0_ + *a*_*n*_*η*. The linear fit parameters are listed in Table S4.

As shown in Fig. 9, the slopes of the relaxation times (*τ*_2_ and *τ*_3_) to η of the positively charged system (aq. ImHCl) were steeper than those of its neutral system (aq. Im). In contrast, the relaxation times of the negatively charged system (aq. NaTr) were less sensitive to *η* than those of its neutral system (aq. Tr). It might be surprising that the positively and negatively charged aromatics in the aqueous solutions displayed different orientational dynamics to *η*. Masuda employed nuclear magnetic resonance measurements to compare the rotational relaxation times of ammonium (cation) and perchlorate (anion) ions in various solvents [97, 98]. The slope of the rotational relaxation time to the rotational time based on the SED model for the ammonium ion was much steeper than that for the perchlorate ion. Thus, the difference in the effects of the positive and negative charges on the orientational relaxation is rather universal whether the solute is aromatic or nonaromatic.

SED equation (Eq. 2) is often modified as follows:

4 $$\tau_{{\text{s}}} = \begin{array}{*{20}c} {\eta V} \\ {\overline{{k_{B} T}} } \\ \end{array} fC,$$

where *f* is the shape factor and *C* is the coupling parameter between the solute and solvent [95, 99]. The quantum chemistry calculations of the four aromatics using the MP2/aug-cc-pVTZ level of theory revealed that the average rotational constant of Im^+^ was smaller than that of Im, whereas that of Tr^−^ was larger than that of Tr (with average rotational constants of 7.698 and 7.984 GHz for Im^+^ and Im, respectively, and 8.623 and 8.386 GHz for Tr^−^ and Tr, respectively). Although the tendencies were identical with the slope of the relaxation times to η, the ratios of the rotational constants of the charged aromatics to those of their neutral analogs were very small compared with the ratios of the slopes obtained in the fs-RIKES experiments. Therefore, the coupling parameter might be crucial to the different *η* dependence of the relaxation times in the four aqueous solution systems. *C* of the stick condition is unity, whereas the slip condition is less than 1 (0 in the case of no frictional rotation of a spherical solute) [100]. Thus, our results indicated that the positively charged aromatics in the aqueous solutions were more in the stick condition than the neutral aromatics in the aqueous solutions; however, the negatively charged aromatics in the aqueous solutions were more in the slip condition than its neutral aromatic in aqueous solutions.

Based on the quantum chemistry calculation results (Figs. 7 and 8), it is plausible that the counterion (Cl^−^) on the Im^+^ ring inhibited its out-of-plane orientational motion. Similar local structures of Im^+^–Cl^−^ (II) (Fig. 7) can be expected to exist at a high concentration. Therefore, we conclude that the local structure of Im^+^ in the aqueous ImHCl solutions is attributed to the intense concentration dependence of the orientational relaxation time.

Regarding the comparison of Tr^−^ and Tr, the NBO analysis results indicated that Tr exhibited a positively charged hydrogen with a large magnitude, while Tr^−^ did not contain such a positively charged hydrogen with a large magnitude (Fig. 8). The quantum chemistry calculation results of the charge populations indicated that Tr could form hydrogen bonding clusters or aggregations between Tr molecules (Fig. 7) at high concentrations. Thus, it is plausible that the orientational relaxation times of the aqueous Tr solutions could be more concentration-dependent than those of the aqueous NaTr solutions.

### Intermolecular vibration

**Fig. 10:**
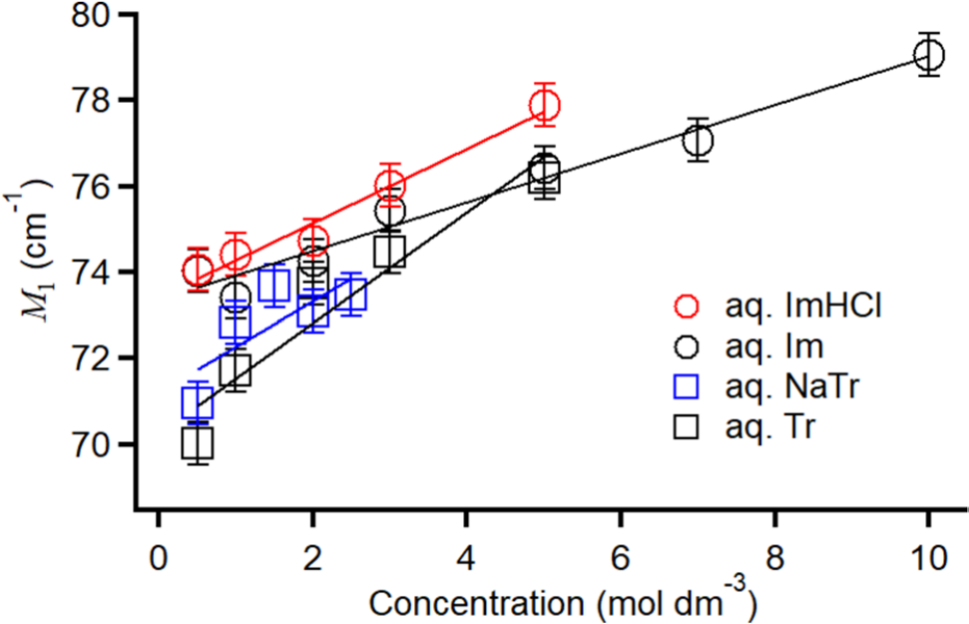
Plots of *M*_1_ of the difference spectrum vs. *c* for the ImHCl (red circles), Im (black circles), NaTr (blue squares), and Tr (black squares) aqueous solutions. Linear fits are also shown

As shown in Fig. 6, the difference spectrum of each aqueous solution system shifted to the higher-frequency region with the increasing concentration. Generally, any intramolecular band of the four explored aromatics is absent in the frequency region of less than 200 cm^–1^ [77], indicating that the broadened band in Fig. 6 is mainly attributed to the intermolecular vibrations. To discuss the concentration dependence of the intermolecular vibrational bands in the four aqueous solution systems, the values of the first moment (*M*_1_) of the broadened bands in the difference spectra were estimated as follows:

5 $$M_{1} = \smallint \omega I\left( \omega \right)d\omega /\smallint I\left( \omega \right)d\omega ,$$

where *I*(*ω*) is the frequency-dependent intensity of the difference spectrum, thus, *M*_1_ expresses the center frequency of the spectrum band. The integral was calculated in the 0–300 cm^−1^ frequency range. Figure 10 shows the *M*_1_ vs. *c* plots for the four aqueous solution systems. The linear fit functions of the four solution systems were given as follows: *M*_1_ = 73.4 + 0.865*c*, 73.4 + 0.565*c*, 71.2 + 1.06*c*, and 70.3 + 1.29*c* for the aqueous solutions of ImHCl, Im, NaTr, and Tr, respectively.

The linear fit results confirmed that the *M*_1_ values extrapolated to *c* = 0 (intrinsic *M*_1_) for the aqueous solutions of ImHCl and Im, and the values for NaTr and Tr were similar, while those of ImHCl and NaTr or Im and Tr were different. This finding indicated that the molecular or ionic shape or geometry is a crucial determinant of the characteristic frequencies of the intermolecular vibrational bands in the aqueous aromatic solutions with a dilute concentration limit.

Zhong and Fourkas have reported that the molecular shape plays a significant role in the spectral-line shape of the low-frequency spectrum in neat aromatic molecular liquids. However, the effects of the local structure and electrostatic forces on the spectrum were relatively minimal [56]. Notably, the characteristic frequency of the low-frequency band in neat aromatic molecular liquids was influenced by the local structure and electrostatic forces [56]. However, in the systems explored in this study (*aqueous* aromatic solutions), the line shapes of the aqueous solutions of the aromatics having the same isoelectronic structures (Im^+^ and Im or Tr^−^ and Tr) were different even in the dilute concentration conditions (< 1.0 mol dm^–3^, Fig. 6). By considering this and the intrinsic *M*_1_, we confirmed that the intermolecular vibrations of aromatics in the aqueous solutions differed from those in neat aromatic liquids.

The librational motion in liquids often discussed with the rotational constants of molecules [78, 101, 102]. The quantum chemistry calculations of the four aromatics at the MP2/aug-cc-pVTZ level of theory revealed that each aromatic exhibited unique average rotational constants (e.g., 7.698, 7.984, 8.623, and 8.386 GHz for Im^+^, Im, Tr^−^, and Tr, respectively, also see Table S3). Although the four aromatics exhibited isoelectronic structures, their molecular shapes were different. Additionally, the aqueous solutions of Im^+^ and Tr^−^ contained counterions. Thus, we expected that the line shapes of the four aqueous solution systems were not the same. Furthermore, we note that the average rotational constants of Im^+^ and Im and Tr^−^ and Tr were close and that their tendencies were close to those of the intrinsic *M*_1_.

Notably, the concentration dependence of *M*_1_ in the present aqueous solution systems is not straightforward to understand. In simple aprotic molecular liquids, *M*_1_ of the low-frequency spectral band, mainly stemming from the intermolecular vibration, was proportional to the square root of *γ* divided by *ρ* ($\sqrt{\gamma /\rho }$) [17]. Although the correlations were weak compared with those of the aprotic molecular liquids, aromatic and nonaromatic ILs also exhibited linear correlations between *M*_1_ and $\sqrt{\gamma /\rho }$ [46, 47]. However, the present aqueous aromatic solutions did not manifest the relationship (our results indicated that *M*_1_ increased with the concentration, whereas $\sqrt{\gamma /\rho }$ decreased). The breakdown of the relation between *M*_1_ and $\sqrt{\gamma /\rho }$ has been observed in an aqueous lidocaine solution [29] and several IL and molecular liquid mixture systems [103–105]. This breakdown can be caused by the microheterogeneities or local structures or preferential solvations of liquids and solutions [57]. The temperature dependence of *M*_1_ to $\sqrt{\gamma /\rho }$ for the aqueous aromatic solutions with a concentration of 5.0 M correlated well with that for aromatic ILs [77], although the concentration dependence of the aqueous solution systems did not exhibit a significant relation between *M*_1_ and $\sqrt{\gamma /\rho }$, indicating that the microenvironment or local structure of the aromatics in the aqueous solution varied with the concentration. In addition to $\sqrt{\gamma /\rho }$, the morment of inertia *I* (or rotational constant) is often compared with the vibrational frequency of the intermolecular vibrational band in liquids [78, 101, 102], since the libration is a rotational vibration. One might think that $\sqrt{\gamma /I}$ can be a good parameter to compare with *M*_1_ if the contribution of the libration to the intermolecular vibrational band is dominant. However, the relation of *M*_1_ to the concentration of the aqueous aromatic solutions (Fig. 10) does not correlate with the the relation of γ with the concentration. Accordingly, the intermolecular vibrataional band of the present aqueous aromatic solutions does not simply correlate with the bulk and molecular parameters because of the microenvironment or local structure of the aromatics.

As shown in Fig. 10, the slope of the plot of *M*_1_ to *c* in the aqueous Im solutions was the mildest among those of the four aqueous solutions. As the aqueous Im solutions exhibited the highest solubility among the present aqueous aromatic solution series, the mild concentration dependence of the intermolecular interaction in this system possibly facilitated the slight concentration dependence of *M*_1_.

Although the concentration range for the aqueous NaTr solutions might be narrow when discussing the concentration dependence of *M*_1_ therein, the comparison between the aqueous NaTr and Tr solutions indicated a similar slope of *M*_1_ to *c*. The similar *M*_1_ slopes to *c* of the aqueous NaTr and Tr solutions indicated that the counterion (Na^+^) did not affect the concentration dependence of the aromatic intermolecular vibrations, and this is dissimilar to the case of the aqueous ImHCl solutions that exhibited the steeper concentration dependence of *M*_1_ in the aqueous Im solutions. The difference in the concentration dependence of the aromatic intermolecular vibrations (mostly ring libration) is attributable to the local structure, including the counterion. Notably, Cl^−^ inhibits imidazolium ring libration compared with the neutral aromatic ring librations in aqueous solution, whereas Na^+^ does not influence the aromatic ring libration.

The quantum chemistry calculations of the clusters (Fig. 7) revealed that Im^+^–Cl^−^ exhibited an out-of-plane stable structure probably attributable to the chemical structure and charge distribution of Im^+^ (Fig. 8). The possibility of an out-of-plane stable structure of Im^+^–Cl^−^ also indicated that the out-of-plane structures of Im^+^ and Cl^−^ could exist in higher-concentration solutions. Conversely, Na^+^–Tr^−^ displayed only in-plane optimized and locally stabilized structures. The existence of a counterion on the aromatic ring restricts aromatic ring libration, except if the counterion is located in the plane. Thus, the *M*_1_ to *c* slope of the aqueous ImHCl solutions was steeper than that of the aqueous Im solutions. However, the aqueous NaTr and Tr solutions obtained similar slopes. Thus, the local structure of the aromatic bearing a counterion in the solution is crucial to determining the concentration dependence of the aromatic ring libration in such a solution. Notably, this applies to a similar concentration dependence of the orientational dynamics in the aqueous ImHCl solutions compared with the aqueous Im solutions discussed in Sect. "Orientational relaxation".

The reason why the concentration dependence of the intermolecular vibrational band in the aqueous NaTr solutions compared with that in the aqueous Tr solutions deviated from the concentration dependence of the orientational dynamics might be surprising. Namely, the concentration dependence of *M*_1_ of the intermolecular vibrational band for the aqueous NaTr solutions was similar to that for the aqueous Tr solutions, although the orientational dynamics in the aqueous Tr solutions exhibited a stronger concentration dependence than that in the aqueous NaTr solutions. In Sect. "Intermolecular vibration", the stronger concentration dependence of the orientational dynamics in the aqueous Tr solutions than in the aqueous NaTr solutions was attributed to the hydrogen bonding cluster formation or aggregation. Explicit charge sites for generating hydrogen bonds (hydrogen-bond donor site) were observed in Tr but not in Tr^−^ (Fig. 8). The hydrogen bonding cluster or aggregation might also inhibit aromatic ring libration in the higher-concentration solutions. Regarding aromatic ring libration, the intermolecular hydrogen bond between the Tr molecules does not significantly affect the aromatic ring libration along the hydrogen bond axis. This accounts for our observation of similar concentration dependence for *M*_1_ of the intermolecular vibrational band in the aqueous NaTr and Tr solutions and a stronger concentration dependence of the orientational dynamics in the aqueous Tr solutions than in the aqueous NaTr solutions.

## Conclusions

We investigated the concentration dependence of the intermolecular vibrations and orientational relaxations in aqueous ImHCl, Im, NaTr, and Tr solutions based on fs-RIKES. To discuss the dynamics as well as the bulk properties, we measured the solutions’ *ρ*, *η*, and *γ*. Further, the picosecond dynamics of the aqueous solutions by fs-RIKES were mainly attributed to the orientational dynamics of the aromatics and analyzed using a triexponential function. The intermediate (*τ*_2_, several picoseconds) and slow (*τ*_3_, 5–35 ps) time constants increased with the increasing solution concentrations. Further, the aqueous ImHCl solutions exhibited a steeper slope of the time constants to *η* compared with the aqueous Im solutions. In contrast, the aqueous NaTr solutions exhibited a milder slope for the time constants to *η* than the aqueous Tr solutions. These findings indicated that the aqueous ImHCl solutions dominated the more stick condition than its neutral aromatic solutions and that the aqueous NaTr solutions dominated the more slip condition than its neutral aromatic solutions. In the aqueous aromatic solutions, the intermolecular vibrational band blueshifted with the increasing concentration, indicating the increasing intermolecular interaction with the increasing concentration; these results did not agree with the bulk parameter, $\sqrt{\gamma /\rho }$. The concentration dependence of *M*_1_ for the aqueous ImHCl solutions was more substantial than that for the aqueous Im solutions, whereas that for the aqueous NaTr solutions was similar to that for the aqueous Tr solutions. The quantum chemistry calculation results of the optimized structures of Im^+^–Cl^−^ and Na^+^–Tr^−^ clusters revealed that the localized structures of charged aromatics could account for the difference in the concentration dependence of *M*_1_ of the intermolecular vibrational bands in the aqueous aromatic solutions.

## Supplementary Information

Below is the link to the electronic supplementary material.

Supplementary material (pdf 554 KB)

## Data Availability

Data will be made available on reasonable request.
