# Supplementary material for: Femtosecond Raman-induced Kerr effect spectroscopic study of the intermolecular dynamics in aqueous solutions of imidazolium hydrochloride, imidazole, sodium triazolide, and triazole: concentration dependence
Source: Anal Sci. 2024 Nov 20;41(3):187–200. doi: 10.1007/s44211-024-00692-7 (PMC11832583; doi:10.1007/s44211-024-00692-7)
Supplement: Supplementary file 1 — Supplementary material (pdf 554 KB) [file 44211_2024_692_MOESM1_ESM.pdf]

Supplementary Information

10.1007/s44211-024-00692-7

Femtosecond Raman-induced Kerr effect spectroscopic study of intermolecular dynamics in aqueous solutions of imidazolium hydrochloride, imidazole, sodium triazolide, and triazole: concentration dependence

Masako Shimizu and Hideaki Shirota*

Department of Chemistry, Chiba University

1-33 Yayoi, Inage-ku, Chiba 263-8522, Japan

E-mail: shirota@faculty.chiba-u.jp

Table S1. Density ρ, Viscosity η, and Surface Tension γ of Aqueous Solutions of ImHCl, Im, NaTr, and Tr at 293 K.

	Conc. (mol dm^{-3})	ρ^{a} (g dm^{-3})	η^{b} (mPa s)	γ^{c} (mN m^{-1})
aq. ImHCl	0.5	1.0125	1.003	72.74
	1.0	1.0262	1.065	71.75
	2.0	1.0521	1.202	70.76
	3.0	1.0791	1.388	71.26
	5.0	1.1272	1.840	71.95
aq. Im	0.5	1.0029	1.048	68.18
	1.0	1.0069	1.104	64.51
	2.0	1.0154	1.257	59.05
	3.0	1.0234	1.377	55.97
	5.0	1.0373	1.746	52.99
	7.0	1.0511	2.141	50.81
	10.0	1.0700	3.248	50.41
aq. NaTr	0.5	1.0219	1.213	72.64
	1.0	1.0430	1.322	71.75
	1.5	1.0637	1.463	72.74
	2.0	1.0844	1.705	73.24
	2.5	1.1044	1.902	73.44
aq. Tr	0.5	1.0064	1.010	69.87
	1.0	1.0134	1.103	66.89
	2.0	1.0285	1.207	63.71
	3.0	1.0405	1.316	61.83
	5.0	1.0701	1.671	61.23
H_2O		0.9984	1.006	73.04

^{a} ±0.1 %, ^{b} ±5 %, ^{c} ±1 %.

Table S2. Multiexponential Fit Parameters for Aq. Aroma Solutions and Neat Water with Various Concentrations.

	Conc. (mol dm^{-3})	a_1	τ_1 (ps)	a_2	τ_2 (ps)	a_3	τ_3 (ps)
aq. ImHCl [a]	0.5	0.00576 ± 0.00037	0.99 ± 0.07	0.00274 ± 0.00033	4.46 ± 0.62	0.00061 ± 0.00049	9.67 ± 2.33
	1.0	0.00766 ± 0.00027	1.06 ± 0.05	0.00421 ± 0.00019	4.51 ± 0.32	0.00149 ± 0.00032	10.3 ± 0.70
	2.0	0.00871 ± 0.00014	1.35 ± 0.04	0.00678 ± 0.00013	4.95 ± 0.18	0.00231 ± 0.00015	15.2 ± 0.40
	3.0	0.01174 ± 0.00023	1.18 ± 0.03	0.00944 ± 0.00008	5.65 ± 0.09	0.00254 ± 0.00007	22.8 ± 0.32
	5.0	0.01234 ± 0.00010	1.50 ± 0.02	0.00922 ± 0.00006	7.36 ± 0.08	0.00375 ± 0.00004	32.7 ± 0.21
aq. Im [a]	0.5	0.00497 ± 0.00035	1.12 ± 0.17	0.00238 ± 0.00040	3.40 ± 0.83	0.00074 ± 0.00035	9.16 ± 1.54
	1.0	0.00640 ± 0.00022	1.12 ± 0.08	0.00462 ± 0.00020	3.65 ± 0.29	0.00107 ± 0.00025	9.38 ± 0.74
	2.0	0.00973 ± 0.00025	1.08 ± 0.04	0.00793 ± 0.00010	4.28 ± 0.11	0.00134 ± 0.00012	12.7 ± 0.47
	3.0	0.01073 ± 0.00021	1.15 ± 0.04	0.00987 ± 0.00013	4.51 ± 0.11	0.00162 ± 0.00014	14.4 ± 0.52
	5.0	0.01520 ± 0.00019	1.16 ± 0.02	0.01032 ± 0.00009	4.95 ± 0.10	0.00323 ± 0.00013	15.2 ± 0.26
	7.0	0.01613 ± 0.00014	1.28 ± 0.02	0.00972 ± 0.00007	5.71 ± 0.09	0.00300 ± 0.00009	18.7 ± 0.27
	10.0	0.01573 ± 0.00013	1.26 ± 0.01	0.00888 ± 0.00006	5.73 ± 0.06	0.00402 ± 0.00004	23.3 ± 0.14

Continued.

	Conc. (mol dm^{-3})	a_1	τ_1 (ps)	a_2	τ_2 (ps)	a_3	τ_3 (ps)
aq. NaTr [a]	0.5	0.01373 ± 0.00911	0.57 ± 0.15	0.00314 ± 0.00036	2.44 ± 0.57	0.00186 ± 0.00063	5.94 ± 0.65
	1.0	0.01053 ± 0.00183	0.71 ± 0.08	0.00480 ± 0.00033	2.92 ± 0.31	0.00270 ± 0.00059	6.25 ± 0.38
	1.5	0.02563 ± 0.00860	0.53 ± 0.06	0.00674 ± 0.00023	3.12 ± 0.16	0.00290 ± 0.00036	7.56 ± 0.32
	2.0	0.00795 ± 0.00092	1.21 ± 0.20	0.00627 ± 0.00095	3.28 ± 0.62	0.00461 ± 0.00074	8.19 ± 0.41
	2.5	0.01383 ± 0.00216	0.77 ± 0.08	0.00971 ± 0.00032	3.76 ± 0.22	0.00316 ± 0.00053	9.43 ± 0.56
aq. Tr [a]	0.5	0.00878 ± 0.00259	0.76 ± 0.18	0.00273 ± 0.00059	2.65 ± 0.65	0.00146 ± 0.00034	7.96 ± 0.73
	1.0	0.00764 ± 0.00106	0.92 ± 0.21	0.00456 ± 0.00095	2.78 ± 0.49	0.00300 ± 0.00030	9.02 ± 0.37
	2.0	0.01170 ± 0.00226	0.86 ± 0.18	0.00776 ± 0.00094	2.97 ± 0.39	0.00537 ± 0.00046	9.84 ± 0.36
	3.0	0.01099 ± 0.00078	1.09 ± 0.18	0.00812 ± 0.00130	3.23 ± 0.41	0.00725 ± 0.00033	11.9 ± 0.25
	5.0	0.01364 ± 0.00047	1.46 ± 0.09	0.0094 ± 0.00061	4.82 ± 0.32	0.00779 ± 0.00023	19.0 ± 0.28
H_2O [b]		0.00468 ± 0.00090	0.75 ± 0.08	0.00065 ± 0.00108	1.28 ± 0.43		

[a] Fit is from 2 ps.

[b] Fit is from 1 ps.

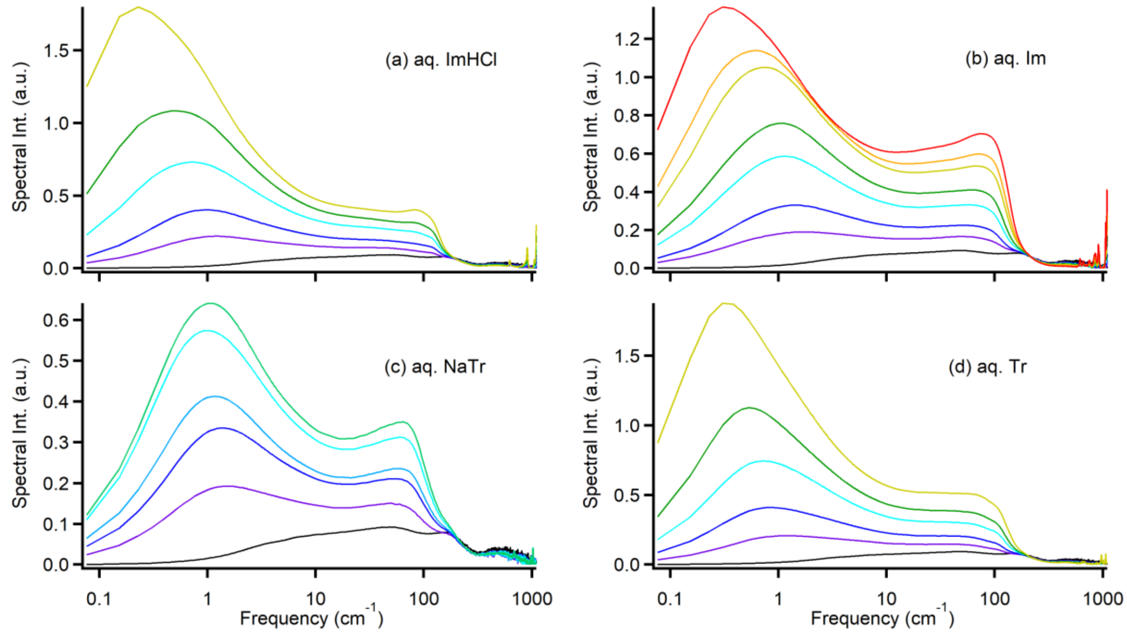

Figure S1. Semilogarithmic plots of broadband Raman spectra of aqueous solutions of (a) ImHCl, (b) Im, (c) NaTr, and (d) Tr with various concentrations (0.5 M: purple; 1.0 M: blue; 1.5 M: light blue; 2.0 M: cyan; 2.5 M: light green; 3.0 M: green; 5.0 M: yellow; 7.0 M: orange; 10.0 M: red).

Table S3. Atom Coordinates at Optimized Structures of Aromatics, Counterions, and Their Clusters at the MP2/aug-cc-pVTZ Level of Theory.

Im^+

Center Number	Atomic Number	Atomic Type	Coordinates (Angstroms) X	Y	Z
1	6	0	0.683333	0.973239	0.000046
2	6	0	-0.684805	0.972236	0.000083
3	6	0	0.000855	-1.141775	-0.000092
4	7	0	1.070603	-0.343338	-0.000022
5	1	0	2.026302	-0.675021	-0.000046
6	1	0	1.388955	1.784030	0.000086
7	1	0	-1.391660	1.781954	0.000154
8	1	0	0.001667	-2.217330	-0.000173
9	1	0	-2.025281	-0.678014	-0.000021
10	7	0	-1.070072	-0.344922	-0.000011

Rotational constants (GHZ): 9.3666553 9.1086627 4.6179288

Im

Center Number	Atomic Number	Atomic Type	Coordinates (Angstroms) X	Y	Z
1	6	0	-0.551111	1.018167	-0.000005
2	6	0	-1.150065	-0.221774	0.000104
3	6	0	0.959420	-0.591153	-0.000128
4	7	0	0.796015	0.761366	0.000028
5	1	0	1.531365	1.448040	0.000022
6	1	0	-0.945826	2.018315	0.000004
7	1	0	-2.202954	-0.445136	0.000196
8	1	0	1.930341	-1.056444	-0.000221
9	7	0	-0.204928	-1.217968	-0.000003

Rotational constants (GHZ): 9.7840208 9.3795787 4.7887660

Tr^-

Center Number	Atomic Number	Atomic Type	Coordinates (Angstroms) X	Y	Z
1	6	0	-1.037206	0.327571	-0.000038
2	6	0	1.037220	0.327536	0.000021
3	7	0	0.000011	1.199430	0.000013
4	1	0	-2.072078	0.641255	-0.000016
5	1	0	2.072086	0.641241	0.000001
6	7	0	0.688029	-0.972086	-0.000077
7	7	0	-0.688054	-0.972078	0.000081

Rotational constants (GHZ): 10.5878795 10.1097130 5.1716364

Tr

Center Number	Atomic Number	Atomic Type	Coordinates (Angstroms) X	Y	Z
1	6	0	1.120270	-0.005501	-0.000011
2	6	0	-0.848739	0.715808	0.000002
3	7	0	0.404452	1.149177	0.000000
4	1	0	2.195606	-0.037189	-0.000009
5	1	0	-1.741806	1.316419	0.000002
6	7	0	-0.859142	-0.633889	-0.000005
7	1	0	-1.643205	-1.265740	-0.000009
8	7	0	0.391864	-1.126050	0.000015

Rotational constants (GHZ): 10.3010029 9.8277437 5.0294049

Im^{+}-Cl^{-} (I)

Center Number	Atomic Number	Atomic Type	Coordinates (Angstroms) X	Y	Z
1	6	0	-2.337043	0.653394	-0.000072
2	6	0	-1.046698	1.128799	-0.000254
3	6	0	-0.898032	-1.023258	-0.000142
4	7	0	-2.218725	-0.713124	0.000070
5	1	0	-2.977922	-1.374227	-0.000100
6	1	0	-3.291243	1.148271	0.000052
7	1	0	-0.708496	2.150130	-0.000358
8	1	0	-0.523149	-2.032155	-0.000077
9	1	0	1.500863	0.066121	-0.000033
10	7	0	-0.164255	0.078613	-0.000258
11	17	0	2.845379	-0.004128	0.000273

Rotational constants (GHZ): 9.6734211 1.0916032 0.9809116

Im^{+}-Cl^{-} (II)

Center Number	Atomic Number	Atomic Type	Coordinates (Angstroms) X	Y	Z
1	6	0	1.620924	0.680441	-0.551809
2	6	0	1.620951	-0.680406	-0.551819
3	6	0	0.101376	-0.000019	0.925775
4	7	0	0.735074	1.073659	0.430190
5	1	0	0.371528	2.005532	0.546501
6	1	0	2.174670	1.382344	-1.145736
7	1	0	2.174719	-1.382280	-1.145760
8	1	0	-0.568124	-0.000041	1.761948
9	1	0	0.371588	-2.005558	0.546455
10	7	0	0.735099	-1.073669	0.430159
11	17	0	-2.051476	-0.000001	-0.324632

Rotational constants (GHZ): 5.7430077 1.8854145 1.7381514

Im^+-Cl^- (II)

Center Number	Atomic Number	Atomic Type	Coordinates (Angstroms) X	Y	Z
1	6	0	-0.261304	0.686392	-0.000162
2	6	0	-0.260438	-0.685232	0.000099
3	6	0	-2.384864	-0.000722	-0.000066
4	7	0	-1.578007	1.070223	0.000275
5	1	0	-1.906034	2.024101	0.000461
6	1	0	0.606791	1.329457	-0.000321
7	1	0	0.608780	-1.326894	0.000117
8	1	0	-3.458463	-0.001378	-0.000125
9	1	0	-1.903485	-2.024951	0.000101
10	7	0	-1.576669	-1.070656	0.000078
11	17	0	2.680869	0.000004	-0.000113
Rotational constants (GHZ):			9.1560844	1.2317638	1.0857045

Na^+-Tr^- (I)

Center Number	Atomic Number	Atomic Type	Coordinates (Angstroms) X	Y	Z
1	6	0	-1.072640	-1.043339	0.000007
2	6	0	-1.072696	1.043318	0.000032
3	7	0	-1.922474	-0.000033	0.000040
4	1	0	-1.388203	-2.074362	-0.000005
5	1	0	-1.388313	2.074324	0.000027
6	7	0	0.223023	0.693691	-0.000021
7	7	0	0.223057	-0.693636	-0.000012
8	11	0	2.362118	0.000001	-0.000028
Rotational constants (GHZ):			10.4690435	2.3767329	1.9369884

Na^+-Tr^- (II)

Center Number	Atomic Number	Atomic Type	Coordinates (Angstroms) X	Y	Z
1	6	0	-0.490104	-1.048784	0.000453
2	6	0	-0.496446	1.050594	0.000518
3	7	0	0.377652	0.003599	0.001297
4	1	0	-0.175217	-2.081911	0.000810
5	1	0	-0.187731	2.085528	0.000915
6	7	0	-1.777041	0.683791	-0.000697
7	7	0	-1.772923	-0.689848	-0.000667
8	11	0	2.589857	0.000248	-0.000643

Rotational constants (GHZ): 10.4400597 2.0195274 1.6921904

Tr-Tr

Center Number	Atomic Number	Atomic Type	Coordinates (Angstroms) X	Y	Z
1	6	0	1.635839	-0.789109	-0.000010
2	6	0	2.595571	1.090910	0.000019
3	7	0	1.383687	0.550406	0.000011
4	1	0	0.852819	-1.529058	-0.000009
5	1	0	2.839997	2.138857	0.000025
6	7	0	3.514375	0.106607	-0.000033
7	1	0	4.519376	0.177308	-0.000063
8	7	0	2.931240	-1.104975	0.000047
9	6	0	-3.026791	-0.951580	-0.000024
10	6	0	-2.635762	1.105866	-0.000013
11	7	0	-3.654237	0.250565	-0.000035
12	1	0	-3.546536	-1.894089	-0.000025
13	1	0	-2.701154	2.180319	-0.000022
14	7	0	-1.468181	0.435529	0.000002
15	1	0	-0.498707	0.767803	0.000010
16	7	0	-1.689591	-0.892083	0.000043

Rotational constants (GHZ): 5.0840702 0.5415320 0.4894030

Table S4. Linear Fit Parameters of τ_2 and τ_3 vs. η for the Aqueous Solutions of ImHCl, Im, NaTr, and Tr.

	$\tau_{2,0}$ (ps)	a_2 (ps mPa^{-1} s^{-1})	$\tau_{3,0}$ (ps)	a_3 (ps mPa^{-1} s^{-1})
aq. ImHCl	0.75 ± 0.20	3.565 ± 0.152	−19.0 ± 2.95	28.56 ± 2.210
aq. Im	2.85 ± 0.47	1.027 ± 0.256	4.09 ± 1.43	6.224 ± 0.773
aq. NaTr	0.60 ± 0.43	1.649 ± 0.280	−0.15 ± 0.77	5.012 ± 0.500
aq. Tr	0.32 ± 0.15	2.243 ± 0.115	−9.25 ± 1.67	16.42 ± 1.300
